# Phosphorylation Modulates Survivin Function in Behcet’s Disease

**DOI:** 10.34172/apb.2020.033

**Published:** 2020-02-18

**Authors:** Yasamin Pahlavan, Naser Samadi, Khalil Ansarin, Alireza Khabbazi

**Affiliations:** ^1^Drug Applied Research Center, Tabriz University of Medical Sciences, Tabriz, Iran.; ^2^Connective Tissue Diseases Research Center, Tabriz University of Medical Sciences, Tabriz, Iran.; ^3^Department of Molecular Medicine, Faculty of Advanced Medical Sciences, Tabriz University of Medical Sciences, Tabriz, Iran.; ^4^Students Research Committee, University of Tabriz Medical Sciences, Tabriz, Iran.; ^5^Department of Biochemistry, Faculty of Medicine, Tabriz University of Medical Sciences, Tabriz, Iran.; ^6^Rahat Breath and Sleep Research Center, Tabriz University of Medical Sciences, Tabriz, Iran.

**Keywords:** Autoimmunity, Apoptosis, Lymphocytes, Inhibition of apoptosis proteins (IAPs), Behcet’s disease

## Abstract

***Purpose:*** Survivin is critical for proliferation, maturation, homeostasis and differentiation of effector and memory lymphocytes. In this study the baculoviral inhibitors of apoptosis proteins (IAPs) repeat containing 5 (BIRC5) mRNA, survivin, and phosphorylated survivin expression were evaluated in peripheral blood mononuclear cells (PBMCs), and plasma of patients with Behcet’s disease (BD).

***Methods:*** In this study, 26 Iranian Azari patients diagnosed with BD and 30 healthy controls were recruited. Total RNA was extracted from PBMCs. The expression level of survivin was measured by quantitative real-time polymerase chain reaction (PCR). Survivin plasma levels were measured using survivin Enzyme-linked immunosorbent assays. Also, western blotting analysis was performed to measure phosphorylated-survivin and survivin levels in PBMCs and plasma of patients with BD.

***Results:*** In a pilot study, we showed that BIRC5 gene expression increased in BD patients compared with healthy controls (*P*<0.05). Western blotting analysis indicated that there was an increase in phosphorylated survivin expression in PBMCs of BD patients. Our data from western blot analysis showed survivin level in plasma samples of BD patients was similar to healthy controls. No significant differences were observed between plasma survivin levels in the BD patients compared with control group (*P*>0.05). The expression of phosphorylated survivin at Thr34 in PBMCs of BD patients with active disease was increased. Plasma phosphorylated survivin levels in having BD patients were also downregulated compared to healthy individuals.

***Conclusion:*** Analysis of PBMCs indicated increasing expression level of phosphorylated survivin in PBMCs of BD patients. There was also a downregulation in phosphorylated survivin levels in plasma of BD patients.

## Introduction

Behcet’s disease (BD) is a vasculitis characterized by recurrent attacks and frequent relapses of manifestations including oral aphthous ulcers, genital sores, skin lesions, arthritis, uveitis, gut and central nervous system involvement with various occurrence in patients.^1^ BD is more distributed in ancient Silk Road countries.^2^ The etiopathology of BD is not completely clear. It is reported that environmental factors including health status of oral and dental, also genital system, stress, smoking, and nutrition have direct correlation with incidence of BD. A line of evidence indicate the familial hereditary basis for BD patients.^3-5^ Deregulated activation of innate immune response including NK cells, γδ T cells and neutrophils are involved in pathogenesis of BD.^1,6^ Defective regulation of apoptosis process has been introduced in the pathogenesis of BD.^7^

Survivin encoded by baculoviral inhibition of apoptosis proteins (IAPs) repeat containing 5 (BIRC5) gene. This gene is located on chromosome 17q25 and have 4 exons and 3 introns, a single BIR domain without C-terminus ring finger domain.^8^ There are several spliced variants encoded by BIRC5 gene including Survivin wild type (142 amino acid), ΔEx3 (137 amino acid), 2B (165 amino acids), 3B (120 amino acid), 2α (74 amino acids), 3α (78 amino acids). Phosphorylation at Thr 34 is necessary for antiapoptotic activity of survivin protein.^9^ High BIRC5 gene expression considered in fetal development and pathologic conditions including tumors and autoreactive cell survival.^8,10,11^ Recently, survivin dysregulation is taken into consideration strongly in prognosis and disease activity of autoimmune diseases.^10,12^ Defective apoptosis is the irrevocable problem in autoimmune diseases.^8^ Survivin is an anti-apoptotic protein expressed in fetal development, tumor cells and immune cells.^10,13^ Survivin is able to inhibit caspase 3 function in apoptosis process by interfering during G2/M phase of mitosis in deregulated cell cycle checkpoints to promote abnormal cell survival.^14^ Studies indicated that posttranslational modifications on survivin like phosphorylation and acetylation have a key role in regulation of cellular functions for survivin. Survivin can phosphorylate in several sites including Thr 34, Thr 53, Thr 117, and Serin 20 and etc.^15^ These modifications provide various molecular functions for survivin. Survivin phosphorylation on Thr34 is regulated by CDK1/P34cdc2 cyclin B1 supported by human cervical adenocarcinoma, oral sub-mucosal fibrosis and oral squamous cell carcinoma studies.^16^ Unlike other IAP family members, survivin has only one BIR domain. Threonine 34 amino acid residue is located in this BIR domain. In fact, recent studies confirmed that phosphorylation of survivin on Thr34 increase cyto-protective effects on cancer cells.^17^

Considering to unknown etiopathogenesis of BD, it is required to identification of specific diagnostic and prognostic markers for disease activity and therapy. Survivin regulates this process under influence of phosphorylation at Thr34 by p34 cdk2 kinase. In this paper, we aimed to evaluate the role of phosphorylated survivin parallel with survivin expression in peripheral blood mononuclear cells (PBMCs) and circulation of BD patients.

## Materials and Methods

### Study design and sample collection

In this study, Iranian Azari patients with diagnosis of BD (n = 26) including 16 patients with active disease and 10 patients with inactive BD and 30 age, sex and ethnically matched healthy controls were recruited in the study. Whole blood sample was collected from both patients and healthy individuals. BD diagnosis was based on International Criteria of Behcet’s Disease.^18^ Cases were called from the BD clinic of Connective Tissue Diseases Research Center at Imam Reza Hospital in Tabriz University of Medical Sciences between February 2018 and December 2018. Excluding criteria were having renal diseases, liver diseases, malignancy, human immunodeficiency virus infection, diabetes mellitus, other inflammatory, and autoimmune diseases and smoking. BD activity was measured using the Behcet’s Disease Current Activity Form (BDCAF), Total Inflammatory Activity Index (TIAI) and Iranian Behcet’s Disease Dynamic Activity Measure (IBDDAM).^19,20^ According to Indexes, patients with BDCAF ≥ 1 classified as active.^19^

### Sampling

Five milliliters of peripheral blood was obtained from the cubital vein of participants and directly transferred into sodium citrate tubes. PBMCs isolated by Ficoll (Lymphodex, Inno-Train, Germany) density-gradient centrifugation (Sigma) according to manufacture instruction. Then PBMCs were stored at -70°C until next step.

### Quantitative real-time PCR

The expression level of survivin was measured by real-time polymerase chain reaction (PCR) (Roche Diagnostics GmbH, Sandhofer Strasse 116, 68305 Mannheim, Germany). Total RNA was extracted from PBMCs by high pure RNA isolation kit (BioFACT^TM^ Total RNA Prep Kit, Korea) according to the manufacturer’s instructions. Amplification of complementary DNA (cDNA) was performed by reverse transcription using the reverse transcription reagent kit (BioFACT, Korea). Glyceraldehyde-3-phosphate dehydrogenase (GAPDH) used as a reference gene. The specific primers and PCR program are survivin: Forward: ATTTGATTCGCCCTCCTCCC, survivin: Reverse: TCCAGAGGTTTCCAGCGAAG, GapDH: Forward: AATGGGCAGCCGTTAGGAAA, GapDH: Reverse: GCCCAATACGACCAAATCAGAG. The ΔΔCt formula was used for calculating the relative expression levels of survivin.

### Enzyme-linked immunosorbent assays (ELISA)

Survivin plasma levels was measured in plasma by Human survivin ZellBio GmbH ELISA kit [Cat. No: ZB-13904C-H9648, Germany] according to the manufacturer’s recommendations.

### Western immunobloting analysis

Cells were harvested, and total protein lysates were resolved. Western blotting analysis performed on all samples from PBMCs and plasma, by anti-phosphorylated-survivin (Thr 34), **(**anti-p-survivin (Thr 34), Cat. No: sc-16320, SANTA CRUZ**)**and survivin antibodies (anti-Survivin, Cat. No: sc-17779, SANTA CRUZ), and anti- β-Actin (Anti-β-Actin, Cat. No, sc-130657, SANTA CRUZ). Proteins were first separated by electrophoresis in an acrylamide gel containing sodium dodecyl sulfate (SDS). After separation, protein bands were transfected on the membrane, then emerged with a little luminescence method and using radiographic film. Band density was measured by ImageJ software. All examinations repeated three times to confirm the validity of the data.

### Statistical analysis

Statistical analysis was performed using SPSS software version 16.0 (SPSS, Chicago, IL, USA). The normal distribution of the data was tested with the Kolmogorov–Smirnov test with Lilliefors correction. The quantitative data were presented as mean ± standard deviation (SD). Differences in the expression levels of survivin in PBMCs and plasma between the groups were analyzed by the Mann Whitney U-test. P-value < 0.05 was considered as statistically significant.

## Results and Discussion

### BIRC5 gene is overexpressed in PBMCs in BD

**Figure 1:**
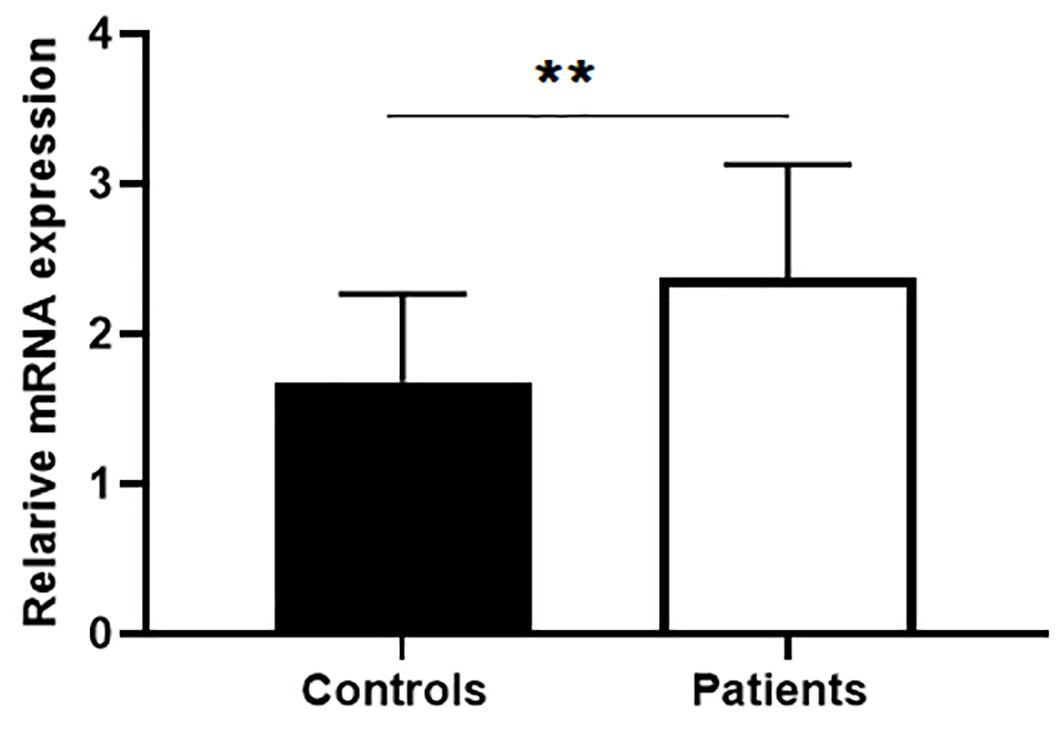
Expression of survivin in peripheral blood cells in Behcet’s patients. Survivin expression significantly increased in peripheral mononuclear cells in Behcet’s patients (n=19) compared with normal control (n=16). Data are represented as mean ± SEM. *P* < 0.05 considered as significance level. The asterisks indicate *P* values. * *P* < 0.01; ** *P* < 0.001; *** *P* < 0.0001. ns: non-significant.

Up-regulation of BIRC5 in autoimmune diseases is reported in recent studies.^4,7,10,21^ High BIRC5 level is found in PBMCs of patients with rheumatoid arthritis (RA),^12^ myasthenia gravis^21^ and multiple sclerosis.^22^ We first analyzed BIRC5 gene expression levels in PBMCs by quantitativePCR analysis. Our data revealed that mRNA expression levels of survivin was significantly higher in BD patients compared to healthy controls (*P* < 0.05, Figure 1).

**Figure 2:**
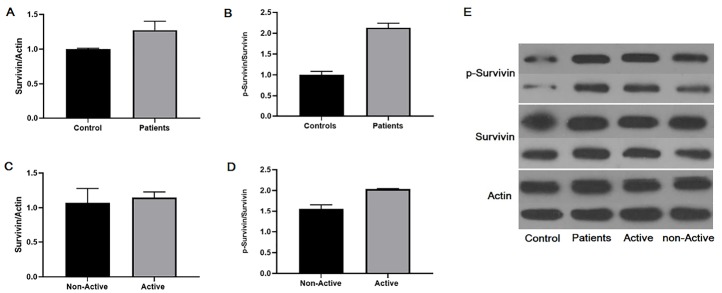
Western blot analysis of survivin and phospho- survivin in peripheral mononuclear cells in patients with Behcet’s disease. Phosphorylation of survivin on Thr34 increased in Behcet’s patients compared with normal controls. A) Survivin expression in peripheral mononuclear cells between Behcet’s patients and control. B) P-survivin expression in peripheral mononuclear cells between Behcet’s patients and control, C) Survivin expression in peripheral mononuclear cells between Behcet’s patients with the active or non-active disease. D) P-survivin expression in peripheral mononuclear cells between Behcet’s patients with the active or non-active disease. E) Gel electrophoresis of survivin and p-survivin expression in studied groups. Data are represented as mean ± SEM. The asterisks indicate *P* values. * *P* < 0.01; ** *P* < 0.001; *** *P* < 0.0001. ns: non-significant.

Because the pattern of mRNA expression varies with protein content and researchers found that RNA may have function without translation to the protein,^23^ we also measured expression of survivin and phosphorylated survivin on Thr34 (p-survivin) in PBMCs by western blot analysis. Therefore, although, analyses showed similar expression patterns of survivin in PBMCs of BD patients and healthy controls (Figure 2A, Figure 2 E. lower panel, Fold of control: 1.36), the expression of phosphorylated survivin at Thr34 was increased in PBMCs of Behcet’s patients. However, these differences were insignificant in the point of statistic (*P* >  0.05, Figure2B, Figure 2E. upper panel, Fold of control: 2.20).

We next classified patients into two groups: patients with active disease and patients with inactive disease. Then, statistical analysis was performed. Our data showed similar expression patterns of survivin in PBMCs between BD patients with active disease and inactive disease (Figure 2C, Figure 2E. lower panel, Fold of control: 1.20), the expression of phosphorylated survivin at Thr34 in PBMCs was tending to increase in Behcet’s patients with active disease (Figure 2D, Figure 2E. upper panel, Fold of control: 2.04). Although, this difference did not reach a statistically significant level (*P* >  0.05).

### Free circulating survivin protein and survivin phosphorylation on Thr34 in BD.

**Figure 3:**
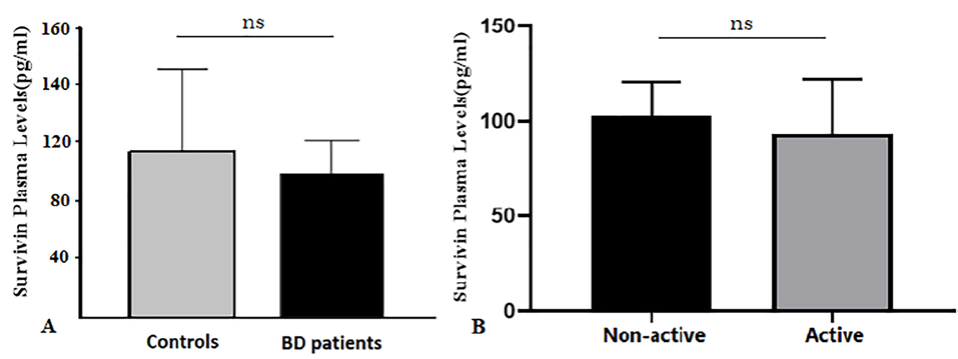
Free circulating of survivin in Behcet’s patients using enzyme linked immunosorbent assay. A) Plasma survivin levels in the BD patients compared with control group. B) Plasma survivin levels in the active BD patients compared with non-active BD patients Data are represented as mean ± SEM. The asterisks indicate *P* values. * *P* < 0.01; ** *P* < 0.001; *** *P* < 0.0001. ns: non-significant.

Previous studies investigated the association between free circulating survivin levels and its impact on disease activity and prognosis in autoimmune diseases and cancers. There is evidence about presence of survivin in circulation. High serum survivin level is reported in other autoimmune diseases including RA^7^ and systemic sclerosis,^4^ and inflammatory arthritis which almost has similar clinical manifestation with BD.^7,24^ Therefore, in this study, we measured survivin and phosphorylated survivin on Thr34 in the plasma samples of BD patients and healthy individuals by western immunoblotting analysis. We next investigated the free circulating survivin in BD patients using enzyme linked immunosorbent assay. No significant differences were observed between plasma survivin levels in the BD patients compared with control group (*P* >  0.05, Figure 3A). This level between BD patients with active and inactive forms of disease was also insignificant (*P* >  0.05, Figure 3B).

**Figure 4:**
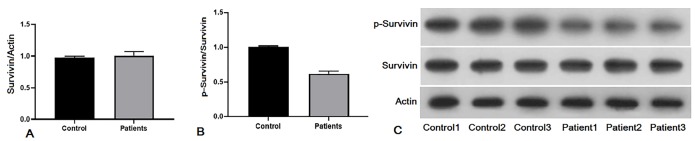
Plasma concentration of survivin and phosphorylated survivin on Thr34 in Behcet’s patients using western blot analysis. A) Survivin plasma level in plasma samples of Behcet’s patients compared with healthy controls. B) P-survivin level of Behcet’s patients compared with healthy controls. C) Gel electrophoresis of western blotting products. There is a trend to decreasing in p-survivin level in plasma of Behcet’s patients. Data are represented as mean ± SEM.

Previous studies performed on autoimmune diseases and cancers investigated the relationship between serum and plasma survivin level and prognosis. Also, our data from western blot analysis showed that the survivin level in plasma samples of Behcet’s patients was similar to healthy controls, (Figure 4A, Figure 4C, lower panel, Fold of control: 1.05). The phosphorylated survivin level was lower in patients with BD. However, these differences were insignificant in the point of statistic (*P* >  0.05, Figure 4B, Figure 4C, upper panel, Fold of control: 0.64). There was considerable down regulation in p-survivin level in BD patients compared with healthy individuals.

In this study, we had shown that survivin levels in PBMCs of Behcet’s patients critically modulated by phosphorylated survivin on Thr34. Other studies indicated that phosphorylation and acetylation, as post-translational modifications, have a key role in regulating different cellular and molecular function for survivin. As regards, survivin overexpression in PBMCs reported in RA, systemic sclerosis, myasthenia gravis.^10^ We also investigated levels of survivin and phosphorylated survivin on Thr34 in plasma samples of Behcet’s patients. The phosphorylated survivin was increased in PBMCs of Behcet’s patients which tended to increase in BD patients with active disease. Also, our data showed that phosphorylated survivin having a downward trend in the circulation of BD patients. Recent studies have identified the presence of survivin in normal and pathologic adult tissues including polymorphonuclear cells,^25^ T cells^26^ and serum of patients with cancer.^27^ These findings suggested that survivin could have different distribution inside and outside the cell which could have undesirable consequences.^28^ Survivin could be inhibited by ubiquitin proteasomal degradation, the X-linked inhibitor of apoptosis (XIAP)-XAF1 complex^29^ and some of medications,^17^ suggesting decreased level of phosphorylated form of survivin in plasma of this patients.

The expression of phosphorylated survivin at Thr34 was higher in PBMCs of BD patients with active disease compared with patients with inactive disease. Phosphosurvivin which is mainly localized in the nucleus, critically regulates survivin levels inside of malignant cells,^17^ suggesting why phosphorylated survivin showed higher level inside the cell and lower level in plasma, respectively. According to gene expression and proteomic data, researchers estimated the possible kinetic parameters about synthesis, stability and degradation of RNA and proteins beside transcriptional and post-transcriptional regulation. Also, depend on stage of disease progression, survivin expression has different distribution. In active status of the disease the expression of survivin may increase, as well as advanced stage of disease, comparable with silenced stage. Regulation of protein concentration is affected by miss folding stress which may show distinct expression patterns inside and outside of the cells.

Autoimmune diseases arise due to cellular and molecular dysregulation of immune system beside genetics and epigenetics dysregulation.^30,31^ The immunopathology and molecular bases of BD were not identified well. Genetic and environmental factors, lymphocytes activation, autoimmunity, and auto-inflammatory process and cell death and survival imbalance involved in BD initiation and progression.^32,33^ For instance, activation of regulatory T cells and decrease of NK cells may be observed in peripheral blood cells in Behcet’s patients which reflects homing of cytotoxic cells toward inflammation through cytokines belong to Th2 subset in Behcet’s patients that results in cytotoxicity during active phase of the disease.^1,10^ Studies reported that activation and proliferation of γδ T cells, which acts against various microbial infections, provide the establishment of BD^1^. There are several evidence of inconsistency in apoptosis of immune cells in the pathogenesis of BD.^17,34^ Levels of apoptosis regulating genes such as Fas/APO1 and Bcl-2 is increased in inflammatory sites in BD.^16,17,35^ Apoptosis associated regulator molecules including Fas antigen (CD95) is identified on lymphocytes in PBMCs of Behcet’s patients.^7^

Extracellular survivin may play a significant role in inflammatory arthritis as previously reported in human leukocytes in autoimmune arthritis context.^11^ In this context, survivin regulates T cell stimulation and skew immune reactions toward Th2 responses,^36,37^ similar to observed phenotype in Behcet’s patients. Survivin is the inhibitor of the apoptosis process which may prevent caspase-dependent pathway in this process. The mechanism of action for survivin is binding and suppressing the caspase 9 in intrinsic pathway of apoptosis followed by inhibition of caspase 3 and 7, which are executive caspases.^31^

Various functions of survivin are originated from different cellular localization, phosphorylation, and acetylation. Protein phosphorylation is involved in cytoprotection, transition from cell cycle,^5^ subcellular localization.^31^ Phosphorylated survivin has a critical role in controlling the cellular stability and expression of apoptosis regulators.^5,35^ Interestingly, evidence showed that survivin has several promitotic and antiapoptotic functions. Some studies mentioned that phosphorylation of survivin on Thr34 by mitotic kinase p34 cdk cyclin B1, is the key step for activation of survivin.^3,35^ Phosphorylated survivin on Thr34 bind to caspase 9 in Hela cells.^35^ Failure of survivin phosphorylation impairs complex formation with caspase 9. Phosphorylation on serine 20 reduces the antiapoptotic role of survivin. Phosphorylation on Thr34 stimulates binding to caspase 9 and regulates the intrinsic pathway of apoptosis. Phosphorylation of survivin on Thr34 by CDK1/P34^cdc2^ cyclin B1was found in human cancer cells.^37^

In this study for a first time, the plasma level of survivin measured in BD patients. Although survivin is found and act in intracellular locations such as mitochondria, cytoplasm, and nucleus of cells, there is some evidence of presence of this molecule in extracellular space and circulation under pathologic conditions. Several studies considered survivin as a valuable serologic marker in RA which can predict the development and response to treatment in early RA and improve RA risk estimation even development of arthralgia in RA patients.^7,24^

Generally, most of the functions of survivin in regulation of inflammation is not well understood. Although, further studies required for understanding the role of survivin in BD, our findings may open a new perspective in BD pathogenesis from the apoptosis and inflammation sight and crosstalk between BD and survival of auto-reactive cells. Further studies should be performed on the function of phosphorylated survivin in cell organelles and circulation in autoimmune diseases.

## Ethical Issues

The research protocol was approved by the Ethics Committee of Tabriz University of Medical Sciences (IR TBZMED.REC.1397.190) and the study was conducted according to the Declaration of Helsinki (2008). Written informed consents were taken from all study participants.

## Conflict of Interest

Authors declare no conflict of interest in this study.

## Acknowledgments

The authors would like to acknowledge Tabriz University of Medical Sciences (TBZMED) for financial support of this project. This is a scientific outcome from thesis number: 59529 registered in Drug Applied Research Center and Faculty of Advanced Medical Sciences of TBZMED as part of a Ph.D. thesis.

## References

[1] Yamamoto JH, Minami M, Inaba G, Masuda K, Mochizuki M (1993). Cellular autoimmunity to retinal specific antigens in patients with Behcet’s disease. Br J Ophthalmol.

[2] Davatchi F, Chams-Davatchi C, Shams H, Shahram F, Nadji A, Akhlaghi M (2017). Behcet’s disease: epidemiology, clinical manifestations, and diagnosis. Expert Rev Clin Immunol.

[3] Altieri DC (2003). Validating survivin as a cancer therapeutic target. Nat Rev Cancer.

[4] Zare Shahneh F, Mohammadian M, Babaloo Z, Baradaran B (2013). New approaches in immunotherapy of behcet disease. Adv Pharm Bull.

[5] Ghotaslou R, Milani M, Akhi MT, Nahaei MR, Hasani A, Hejazi MS (2013). Diversity of Helicobacter pylori cagA and vacA genes and its relationship with clinical outcomes in Azerbaijan, Iran. Adv Pharm Bull.

[6] Mohammad Hosseini A, Majidi J, Baradaran B, Yousefi M (2015). Toll-like receptors in the pathogenesis of autoimmune diseases. Adv Pharm Bull.

[7] Hamzaoui K, Hamzaoui A, Zakraoui L, Chabbou A (1999). Expression of Bcl-2 in inflammatory sites from patients with active Behcet’s disease. Mediators Inflamm.

[8] Garg H, Suri P, Gupta JC, Talwar GP, Dubey S (2016). Survivin: a unique target for tumor therapy. Cancer Cell Int.

[9] Sampath J, Pelus LM (2007). Alternative splice variants of survivin as potential targets in cancer. Curr Drug Discov Technol.

[10] Pahlavan Y, Kahroba H, Samadi N, Karimi A, Ansarin K, Khabbazi A (2019). Survivin modulatory role in autoimmune and autoinflammatory diseases. J Cell Physiol.

[11] Gravina G, Wasén C, Garcia-Bonete MJ, Turkkila M, Erlandsson MC, Töyrä Silfverswärd S (2017). Survivin in autoimmune diseases. Autoimmun Rev.

[12] Chun-Lai T, Murad S, Erlandsson MC, Hussein H, Sulaiman W, Dhaliwal JS (2015). Recognizing rheumatoid arthritis: oncoprotein survivin opens new possibilities: a population-based case-control study. Medicine (Baltimore).

[13] Altieri DC (2008). Survivin, cancer networks and pathway-directed drug discovery. Nat Rev Cancer.

[14] Altieri DC (2003). Survivin and apoptosis control. Adv Cancer Res.

[15] Cheung CH, Huang CC, Tsai FY, Lee JY, Cheng SM, Chang YC (2013). Survivin - biology and potential as a therapeutic target in oncology. Onco Targets Ther.

[16] Zhou S, Li L, Jian X, Ou X, Jiang H, Yao Z (2008). The phosphorylation of survivin Thr34 by p34cdc2 in carcinogenesis of oral submucous fibrosis. Oncol Rep.

[17] Wall NR, O’Connor DS, Plescia J, Pommier Y, Altieri DC (2003). Suppression of survivin phosphorylation on Thr34 by flavopiridol enhances tumor cell apoptosis. Cancer Res.

[18] (2014). The International Criteria for Behçet’s Disease (ICBD): a collaborative study of 27 countries on the sensitivity and specificity of the new criteria. J Eur Acad Dermatol Venereol.

[19] Shahram F, Khabbazi A, Nadji A, Ziaie N, Banihashemi AT, Davatchi F (2009). Comparison of existing disease activity indices in the follow-up of patients with Behçet’s disease. Mod Rheumatol.

[20] Lawton G, Bhakta BB, Chamberlain MA, Tennant A (2004). The Behçet’s disease activity index. Rheumatology (Oxford).

[21] Kusner LL, Ciesielski MJ, Marx A, Kaminski HJ, Fenstermaker RA (2014). Survivin as a potential mediator to support autoreactive cell survival in myasthenia gravis: a human and animal model study. PloS One.

[22] Sharief MK, Noori MA, Douglas MR, Semra YK (2002). Upregulated survivin expression in activated T lymphocytes correlates with disease activity in multiple sclerosis. Eur J Neurol.

[23] Montazersaheb S, Hejazi MS, Nozad Charoudeh H (2018). Potential of peptide nucleic acids in future therapeutic applications. Adv Pharm Bull.

[24] Erlandsson MC, Turkkila M, Siljehult F, Pullerits R, Eriksson C, Rantapää-Dahlqvist S (2018). Survivin improves the early recognition of rheumatoid arthritis among patients with arthralgia: a population-based study within two university cities of Sweden. Semin Arthritis Rheum.

[25] Altznauer F, Martinelli S, Yousefi S, Thürig C, Schmid I, Conway EM (2004). Inflammation-associated cell cycle-independent block of apoptosis by survivin in terminally differentiated neutrophils. J Exp Med.

[26] Xing Z, Conway EM, Kang C, Winoto A (2004). Essential role of survivin, an inhibitor of apoptosis protein, in T cell development, maturation, and homeostasis. J Exp Med.

[27] Khan S, Jutzy JM, Valenzuela MM, Turay D, Aspe JR, Ashok A (2012). Plasma-derived exosomal survivin, a plausible biomarker for early detection of prostate cancer. PLoS One.

[28] Li F, Yang J, Ramnath N, Javle MM, Tan D (2005). Nuclear or cytoplasmic expression of survivin: what is the significance?. Int J Cancer.

[29] Arora V, Cheung HH, Plenchette S, Micali OC, Liston P, Korneluk RG (2007). Degradation of survivin by the X-linked inhibitor of apoptosis (XIAP)-XAF1 complex. J Biol Chem.

[30] Davatchi F, Shahram F, Chams-Davatchi C, Shams H, Nadji A, Akhlaghi M (2010). Behcet’s disease in Iran: analysis of 6500 cases. Int J Rheum Dis.

[31] Sharifi S, Barar J, Hejazi MS, Samadi N (2015). Doxorubicin changes Bax/Bcl-xL ratio, caspase-8 and 9 in breast cancer cells. Adv Pharm Bull.

[32] Keino H, Okada AA (2007). Behçet’s disease: global epidemiology of an Old Silk Road disease. Br J Ophthalmol.

[33] Mahr A, Belarbi L, Wechsler B, Jeanneret D, Dhote R, Fain O (2008). Population-based prevalence study of Behcet’s disease: differences by ethnic origin and low variation by age at immigration. Arthritis Rheum.

[34] Zhai S, Senderowicz AM, Sausville EA, Figg WD (2002). Flavopiridol, a novel cyclin-dependent kinase inhibitor, in clinical development. Ann Pharmacother.

[35] O’Connor DS, Grossman D, Plescia J, Li F, Zhang H, Villa A (2000). Regulation of apoptosis at cell division by p34cdc2 phosphorylation of survivin. Proc Natl Acad Sci U S A.

[36] Khan S, Bennit HF, Wall NR (2015). The emerging role of exosomes in survivin secretion. Histol Histopathol.

[37] Pannone G, Bufo P, Serpico R, Rubini C, Zamparese R, Corsi F (2007). Survivin phosphorylation and M-phase promoting factor in oral carcinogenesis. Histol Histopathol.

